# Recurrence of Invasive Cervical Resorption after six years of Nonsurgical and Surgical management by Bioceramic-Material

**DOI:** 10.12669/pjms.39.3.6959

**Published:** 2023

**Authors:** Mustafa Hussein AlAttas, Hadeel Yaseen Edrees, Syed Fareed Mohsin, Muhammad Qasim Javed

**Affiliations:** 1 Mustafa Hussein AlAttas, Assistant Professor, Department of Conservative Dental Sciences and Endodontics, College of Dentistry,Qassim University, Buraydah, 52571, Qassim Saudi Arabia.; 2 Hadeel Yaseen Edrees, Assistant Professor, Endodontic Department, Faculty of Dentistry, King Abdulaziz University, Saudi Arabia; 3 Syed Fareed Mohsin, Associate Professor, Department of Oral Maxillofacial Surgery and Diagnostic Sciences, College of Dentistry in Ar Rass, Qassim University, Ar Rass, Saudi Arabia; 4 Muhammad Qasim Javed, Associate Professor, Department of Conservative Dental Sciences and Endodontics, College of Dentistry, Qassim University, Buraydah, 52571, Qassim, Saudi Arabia

**Keywords:** Case Report, Dental Materials, Endodontics, Treatment Outcome, External cervical root resorption

## Abstract

Invasive cervical resorption (ICR) is a phenomenon of unknown etiology that results in the loss of hard dental tissue. To have a successful outcome for a tooth affected by ICR, correct diagnosis and management are needed. With the introduction of new biocompatible materials and the advancement of CBCT imaging, these pathologies can be identified and treated with precision, resulting in promising outcomes. This case report aims to present the management of maxillary central incisors diagnosed with external ICR, treated with bioceramic root repair material, and followed-up to six years.

## INTRODUCTION

Root resorption is the loss of hard dental tissue (cementum and dentin) due to odontoclastic activity. Root resorption can be classified, based on its proximity to the root surface, as internal or external resorption. External cervical resorption is the type of root resorption that initiates on the external surface of a tooth at the cervical area and causes the replacement of mineralized structure with fibrovascular tissue or fibro-osseous tissue. The rapid loss of cementum and dentine in an invasive manner necessitates careful management to arrest the resorptive process. Understanding the etiology and pathogenesis of such a type of resorption is very important for its management. Pathologic tooth resorption is classified into external resorption and internal resorption. External resorption is further classified into external inflammatory resorption, external cervical resorption (ECR), external replacement resorption, and transient apical breakdown.^1^

Due to its unclear etiology, several terms have been used to describe ECR such as invasive cervical resorption (ICR)^2^, subepithelial external root resorption^3^, peripheral inflammatory root resorption^4^, odontoclastoma^5^, and idiopathic external resorption.^6^ The absence/loss of unmineralized predentine or precementum layers is a prerequisite for the onset of the resorptive process. This allows the odontoclastic cells to attach to the hard dental structures and commence resorption after being activated.^4^,^7^,^8^ The anatomic structure of some teeth at the cervical area may lack such coverage, making it prone to odontoclastic resorptive activity.^9^ The activation process of the odontoclasts which start the resorption is still unclear. Some researchers advocate that infection of the sulcus at the cervical area initiates an inflammatory reaction that stimulates the odontoclasts to initiate resorption.^3^,^10^,^11^ In contrast, others attribute it to a non-inflammatory mechanism and suggest that a proliferative fibrovascular/fibro-osseous disorder is responsible for the defect which could be secondarily colonized by microorganisms.^5^,^6^,^12^ Some predisposing factors that are possibly associated with ECR are trauma, orthodontic treatment,^13^ intra-coronal bleaching,^14^,^15^ and periodontal treatment.^10^

Heithersay et al. classified ECR into four classes. Class one describes a small invasive lesion located cervically, Class two designates a well-defined invasive resorptive lesion penetrating close to the coronal pulp, Class three a more profound invasion of coronal dentine, and coronal one-third of the root, and lastly, Class four defines a large invasive resorptive lesion extending beyond the coronal third of the root.^2^ Diagnosis of ECR is challenging clinically since the condition is usually painless and discovered accidentally on radiographs. It is worth mentioning that a pinkish hue of the cervical third of the tooth crown, which has traditionally been considered an indicator of internal root resorption, may also be a sign of external cervical resorption.^12^ This discoloration is caused by the vascular granulation tissue showing through resorbed and thinned tooth structure. Extensive clinical and radiographic investigation of the origin and location of the defect, condition of the dental pulp, and status of the periapical tissues is necessary to determine the type, behavior, and severity of the resorption and to devise the proper treatment plan for a such lesion.

Various bioceramic materials have been used in the management of cervical resorption, such as mineral trioxide aggregate (MTA), glass-ionomer cement (GIC), and calcium-enriched mixture (CEM).^16^ In the present case, the material used was total fill putty (FKG Dentaire SA, Switzerland), a pre-mixed bioceramic, ready to use in endodontic surgery. It comprises calcium silicates, monobasic calcium phosphate, zirconium oxide, tantalum oxide, reinforcing agents, and coagulation agents. Manufacturers claim that the material is biocompatible, hydrophilic, has high pH13,^12^ does not discolor, and has 30 minutes of working time. The material showed similar biocompatibility as MTA.^17^

This case report describes the non-surgical and surgical management of external cervical resorption affecting upper central incisors with six years of follow-up clinically and radiographically by CBCT.

## CASE REPORT

**Fig.1:**
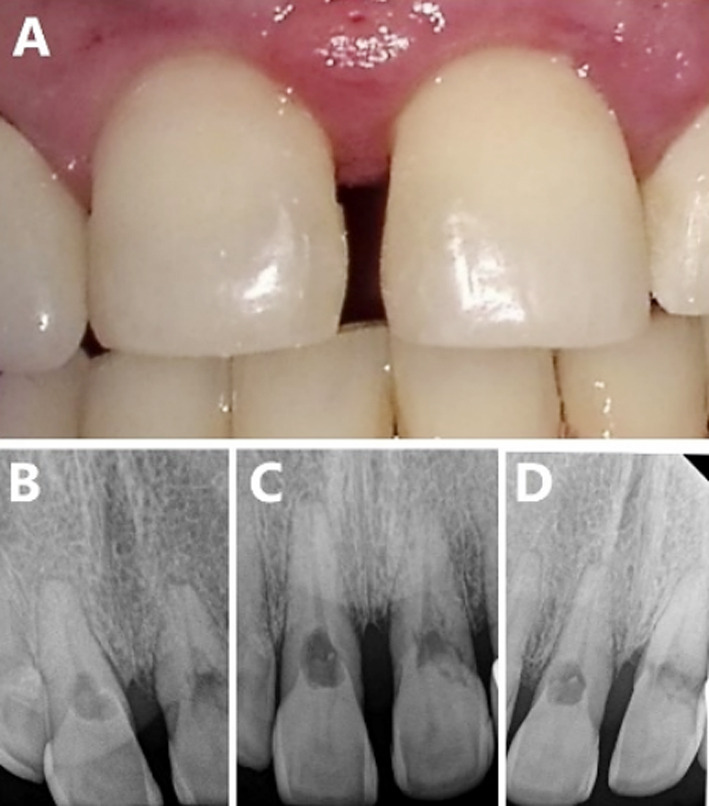
(A) Clinical appearance of Maxillary central incisors, Periapical Radiographs (PAR) (B) Mesially shifted PAR, (C) PAR, (D) Distally shifted PAR.

A 55-year-old female patient presented at the University dental clinic with the chief complaint of generalized bleeding from gums and bad breath. The medical history of the patient was non-contributory. The patient had undergone orthodontic treatment 18 years ago. She had also received multiple restorations in the past. The extra-oral examination didn’t reveal any deviation from normality. Generalized bleeding on probing with clinical attachment loss was noted on intraoral examination. The radiographic examination suggested generalized bone loss. The periodontal diagnosis was stage two with grade two periodontal disease. Additionally, during the radiographic examination, areas of rarefaction were noticed in the cervical areas of upper central incisors on periapical radiographs (Fig.1C) After the dental prophylaxis, the patient was referred to the department of periodontics and endodontic specialty clinic for further investigation and management.

The endodontist examined the upper central incisors. Both central incisors were of normal color and responded normally to cold test. Furthermore, no discomfort, sensitivity, and swelling were noticed on percussion and palpation test (Fig.1A). Probing depth of 5mm was noted on the palatal aspects of both central incisors. Subsequently, two additional radiographs were taken with mesial and distal shifts.

**Fig.2:**
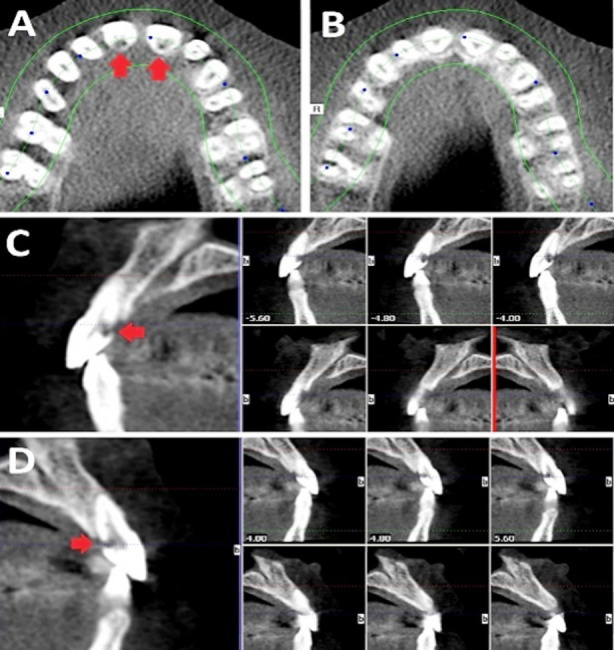
Pre-operative CBCT image, red arrows showing the location of cervical resorption (A) Transverse section at cervical region, (B) Transverse section at mid-root region, (C) Sagittal sections of tooth #11, (D) Sagittal sections of tooth #21.

The shifted radiographs showed that the lesion projection remained at its position in relation to the canal and the outline of the canal was not disturbed (Fig.1B and Fig.1D). For further radiographic examination, cone-beam computed tomography (CBCT) (Care stream, Atlanta, GA) was used. During the radiographic interpretation, both axial and sagittal sections showed radiolucent resorptive lesions cervically at the palatal aspects of central incisors, extending from the external palatal surface and penetrating to perforate the pulp space (Fig.2). The diagnosis for both central incisors was asymptomatic irreversible pulpitis with normal apical tissues and ECR. The patient was informed of the diagnosis and an explanation was provided for all treatment options along with their pros and cons. Subsequently, informed consent was obtained from the patient for carrying out non-surgical endodontic treatment with the surgical management of palatal ECR for central incisors.

**Fig.3:**
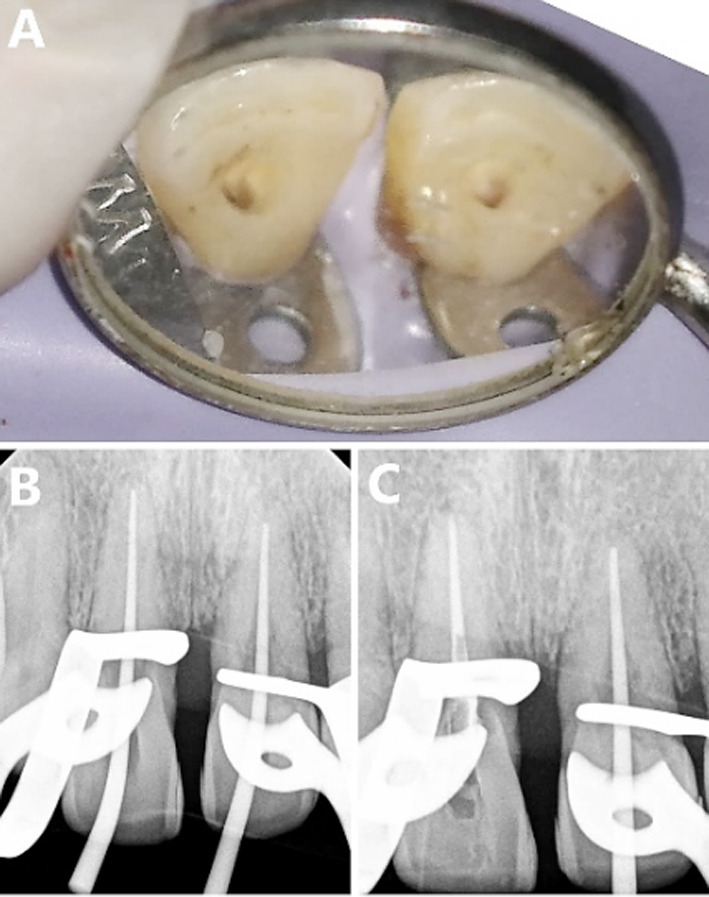
Non-Surgical Endodontic phase (A) Central incisors’ access cavities, (B) Master cone PAR, (C) Apical obturation #11, and canal space preservation for #21.

Two percent lidocaine with 1:100,000 epinephrine (Septodont) was administered as local anesthesia by buccal infiltration technique. Rubber dam isolation was performed for the maxillary central incisors. Teeth #11 and #21 were accessed, cleaned, and shaped using 2.5% sodium hypochlorite as irrigant and ProTaper Next (Dentsply, Switzerland) up to file size X5. After drying the canals with paper points, obturation of the apical portion of tooth #11 was achieved by continuous wave technique by System B using gutta-percha and zinc oxide sealer (Tubli Seal, Kerr**)**. In contrast, due to patient’s time constraints, for tooth #21 the pulp space was maintained by a single cone of X5 to be obturated after surgery. The access cavities for both central incisors were sealed by temporary restorations Cavit (3M, ESPE, USA) (Fig.3).

**Fig.4:**
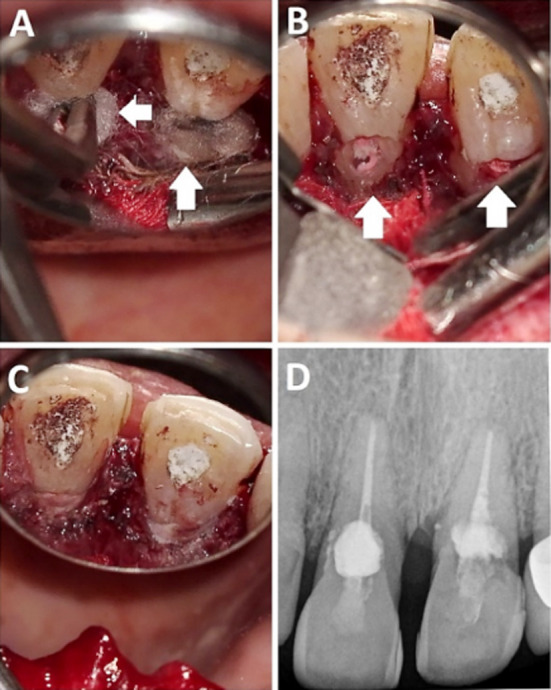
Surgical Management of resorptive defects (A) TCA, soaked cotton pellet on the defect surfaces (DS), (B) The DS after granulation tissue removal (C) DS restoration by a putty bio-ceramic restorative material, D: Post-surgical radiograph.

On the day of surgery, following the administration of 2% lidocaine with 1:100,000 epinephrine (Septodont) by infiltration technique and incisive nerve block, a palatal sulcular incision was performed from the right canine to the left canine. A flap was reflected to expose the cervical area of the palatal surfaces of central incisors. Under magnification (OPMI Carl Zeiss, Germany), the granulation tissue was removed from both teeth, and the defect surfaces were excavated and smoothened with a round bur. After that, a cotton pellet soaked in 90% Trichloracetic acid (TCA) was applied to the defect for five minutes. Following the surface treatment by TCA, the necrotic tissue was removed, and the cavity was excavated with a round bur (Maillefer, Dentsply, Switzerland). Afterward, the defect cavities were filled and restored with a Putty Bioceramic restorative material (FKG, Switzerland). Finally, the flap was repositioned, and interrupted sutures were placed. The temporary filling of tooth #21 was removed for final obturation by continuous wave technique followed by injectable thermoplasticized gutta-percha backfilling the coronal canal space. The patient was referred to the restorative department for final coronal restoration (Fig.4). Subsequently, the patient was requested to come for clinical and radiographic examinations at six and 12 months after treatment. However, the patient failed to show up.

**Fig.5:**
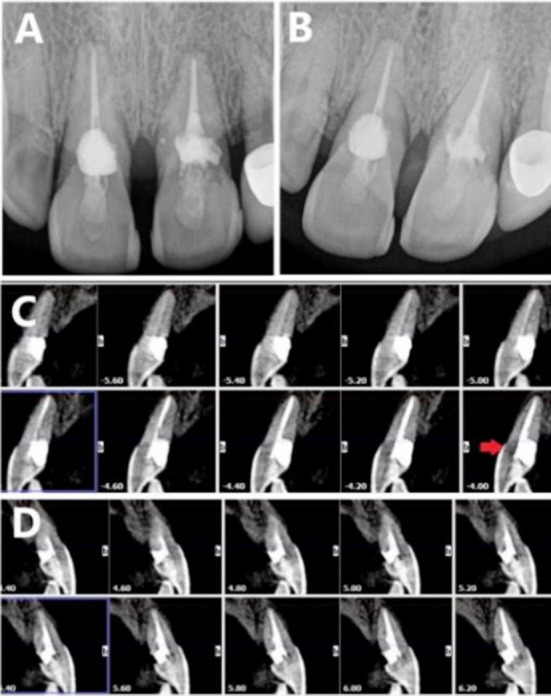
Follow-up radiographs after six years (A) Straight PAR, (B) Distally shifted PAR, (C) CBCT sagittal sections #11, red arrow showing new resorptive activity, (D) CBCT sagittal sections of #21.

The patient presented after six years to seek treatment for another distant symptomatic tooth. At this appointment, the patient’s maxillary incisors were re-evaluated clinically and radiographically, six years post-treatment. (Fig.5). The teeth were asymptomatic to percussion and palpation, and radiographs showed normal apical bone. The CBCT revealed that tooth #21 had an intact structure and surrounding bone, but unexpectedly, tooth #11 had a new area of cervical radiolucency located labial to the canal indicating new resorptive activity (Fig.5C). The patient was informed about the new resorptive lesion and possible treatment options were explained to her.

## DISCUSSION

This report described the management of ECR commencing from examination and diagnosis to treatment and restoration of the resorptive defect. It has been known that ECR is not easily recognized since most of the cases are asymptomatic like the present case where the pulp responded normally to cold test with no pain or tenderness on percussion and palpation. The accidentally discovered resorptive radiolucent lesion over the canal on a periapical radiograph can be differentially diagnosed as external resorption or internal resorption. Since each has a different origin and pathogenesis, it is crucial to identify the type of resorption present in order to manage the case properly.

A radiographic assessment was necessary for ICR diagnosis. Considering the difficulty in differentiating the ICR lesions from internal root resorption, a CBCT image was taken. CBCT offers a superior imaging technique that allows a better understanding of the lesion behavior and aids in diagnosing the exact type of resorption through a 3-D assessment.^18^ The ICR lesion in the current case was classified as Heithersay Class III based on its location and extent as seen on the CBCT scan. The pinkish color indicating internal resorption or ECR^12^ was not seen in our case, which led to the delayed diagnosis of the ICR lesion. This can be explained by the palatal location of the resorptive defect. The labial walls of the crowns were of appropriate thickness, obscuring the color of the granulation tissue. The loss of predentine or precementum layers is required for the resorption to occur.^4^,^7^,^8^ The upper central incisors are primarily affected by ECR. The findings in our case are consistent with other studies that show a greater frequency of ECR in upper incisors, upper canines, and first mandibular molars.^2^

Regarding ICR treatment, proper management of each case is essentially related to its etiology. In this case, the patient had a previous history of orthodontic treatment for correction of inclination of anterior teeth and closure of diastema. Orthodontic treatment is found to be one of the most significant etiological variables, causing 24.1% of ICR lesions.^2^

Several authors have proposed various treatment methods for ICR. The primary goal of ICR treatment is to remove resorptive tissue altogether and to restore the damaged area. The present case depicts a cervical resorptive defect that needed root canal treatment followed by sealing of the resorptive site with Bioceramic using a surgical approach. Different materials can be used for the restoration of defects, such as MTA, resin-modified GIC, and CEM.^16^,^19^-^21^ In this case, a bioceramic material total fill putty (FKG, Switzerland) was used for restoring the palatal defects resulting from the ICR lesion. Bioceramic restorative material has been investigated and showed favorable results in several studies of periodontal cell growth and attachment, making it the material of choice for management in our case.^22^,^23^

The success rate of management of ECR differs depending upon the degree of resorption. Heithersay reported a 77% success rate of Class three lesions if all factors such as resorption control, periapical changes, bone loss, and extraction have been evaluated^12^. He also reported 1.6% recurrence or continuous resorption of class three cases and attributed this finding to the development of new resorptive activity adjacent to or distant from the original site due to incomplete inactivation of resorptive tissue in deeply penetrating channels which are the features of this type of resorption. This explains the treatment outcome of the current case in which after six years tooth #11 showed a recurrence of resorptive activity on the labial surface, away from the site of the original defect which was located lingually close to the cingulum. In contrast, the other tooth #21 responded favorably to the treatment with intact remaining structure and normal surrounding bone.

## CONCLUSION

The findings of this report emphasize the importance of longer follow-up, using CBCT for monitoring the outcome of ECR as well as proper diagnosis and preoperative assessment to establish a comprehensive treatment strategy aiming to arrest the resorptive process and prevent recurrence. Moreover, the utilization of contemporary management strategies including Dental Operating Microscope and TCA/ bioceramic material did not guarantee the prevention of ICR recurrence. This highlights the need for further research to look for more effective methods for the management of the disease.

### Authors’ Contribution:

**MQJ** is responsible for the integrity of the work.

All authors have contributed significantly towards the manuscript, and all authors are in agreement with the manuscript.

## References

[1] Patel S, Ford TP (2007). Is the resorption external or internal?. Dent Update.

[2] Heithersay GS (1999). Clinical, radiologic, and histopathologic features of invasive cervical resorption. Quintessence Int.

[3] Trope M (2002). Root resorption due to dental trauma. Endod topics,.

[4] Gold SI, Hasselgren G (1992). Peripheral inflammatory root resorption:a review of the literature with case reports. J Clin Periodontol.

[5] Fish EW (1941). Benign Neoplasia of Tooth and Bone. J R Soc Med.

[6] Williams RA (1961). Basic periodontology|, Williams &Wilkins Company.

[7] Wedenberg C, Lindskog S (1987). Evidence for a resorption inhibitor in dentin. Eur J Oral Sci.

[8] Wedenberg C (1987). Evidence for a dentin-derived inhibitor of macrophage spreading. Eur J Oral Sci.

[9] Neuvald L, Consolaro A (2000). Cementoenamel junction:microscopic analysis and external cervical resorption. J Endod.

[10] Tronstad L (1988). Root resorption-etiology, terminology and clinical manifestations. Dent Traumatol.

[11] Fuss Z, Tsesis I, Lin S (2003). Root resorption-diagnosis, classification and treatment choices based on stimulation factors. Dent Traumatol.

[12] Heithersay GS (2004). Invasive cervical resorption. Endod topics.

[13] Heithersay GS (1999). Invasive cervical resorption:an analysis of potential predisposing factors. Quintessence int.

[14] Heithersay GS, Dahlstrom SW, Marin PD (1994). Incidence of invasive cervical resorption in bleached root-filled teeth. Aus Dent J.

[15] Harrington GW, Natkin E (1979). External resorption associated with bleaching of pulpless teeth. J Endod.

[16] Utneja S, Nawal RR, Talwar S, Verma M (2015). Current perspectives of bio-ceramic technology in endodontics:calcium enriched mixture cement-review of its composition, properties and applications. Restor Dent Endod.

[17] Saxena P, Gupta SK, Newaskar V (2013). Biocompatibility of root-end filling materials:recent update. Restor Dent Endod.

[18] Patel S, Dawood A, Wilson R, Horner K, Mannocci F (2009). The detection and management of root resorption lesions using intraoral radiography and cone beam computed tomography–an in vivo investigation. Int Endod J.

[19] Ikhar A, Thakur N, Patel A, Bhede R, Patil P, Gupta S (2013). Management of external invasive cervical resorption tooth with mineral trioxide aggregate:A case report. Case Rep Med.

[20] Subramanyappa SK, Parthasarathy B, Manjegowda PG, Rajeev S (2012). Management of perforating invasive cervical resorption:Two case reports. J Indian Acad Oral Med Radiol.

[21] Kusgoz A, Yildirim T, Alp CK, Tanriver M (2017). Management of root resorption with mineral trioxide aggregate complicated by a luxation injury:report of a case with six-year follow-up. J Pak Med Assoc.

[22] Papadopoulou C, Georgopoulou M, Karoussis I, Kyriakidou K, Papadopoulos T (2020). In vitro Evaluation of Biocompatibility and Cytotoxicity of Total Fill Bioceramic Root Repair material putty for endodontic use. Br J Med Health Res.

[23] Rodríguez-Lozano FJ, López-García S, García-Bernal D, Pecci-Lloret MR, Guerrero-Gironés J, Pecci-Lloret MP, Lozano A, Llena C, Spagnuolo G, Forner L (2020). In vitro effect of putty calcium silicate materials on human periodontal ligament stem cells. Applied Sci.

